# Acid Assisted Organosolv Delignification of Beechwood and Pulp Conversion towards High Concentrated Cellulosic Ethanol via High Gravity Enzymatic Hydrolysis and Fermentation

**DOI:** 10.3390/molecules23071647

**Published:** 2018-07-05

**Authors:** Konstantinos G. Kalogiannis, Leonidas Matsakas, James Aspden, Angelos A. Lappas, Ulrika Rova, Paul Christakopoulos

**Affiliations:** 1 Chemical Process and Energy Resources Institute (CPERI), Centre for Research and Technology Hellas (CERTH), 6th km Harilaou-Thermi Rd, 57001 Thessaloniki, Greece; angel@cperi.certh.gr; 2 Biochemical Process Engineering, Division of Chemical Engineering, Department of Civil, Environmental and Natural Resources Engineering, Luleå University of Technology, 971-87 Luleå, Sweden; jamesaspden95@gmail.com (J.A.); ulrika.rova@ltu.se (U.R.); paul.christakopoulos@ltu.se (P.C.)

**Keywords:** beechwood, organosolv delignification, ethanol fermentation, enzymatic hydrolysis, high gravity

## Abstract

Background: Future biorefineries will focus on converting low value waste streams to chemical products that are derived from petroleum or refined sugars. Feedstock pretreatment in a simple, cost effective, agnostic manner is a major challenge. Methods: In this work, beechwood sawdust was delignified via an organosolv process, assisted by homogeneous inorganic acid catalysis. Mixtures of water and several organic solvents were evaluated for their performance. Specifically, ethanol (EtOH), acetone (AC), and methyl- isobutyl- ketone (MIBK) were tested with or without the use of homogeneous acid catalysis employing sulfuric, phosphoric, and oxalic acids under relatively mild temperature of 175 °C for one hour. Results: Delignification degrees (DD) higher than 90% were achieved, where both AC and EtOH proved to be suitable solvents for this process. Both oxalic and especially phosphoric acid proved to be good alternative catalysts for replacing sulfuric acid. High gravity simultaneous saccharification and fermentation with an enzyme loading of 8.4 mg/g_solids_ at 20 wt.% initial solids content reached an ethanol yield of 8.0 *w*/*v*%. Conclusions: Efficient delignification combining common volatile solvents and mild acid catalysis allowed for the production of ethanol at high concentration in an efficient manner.

## 1. Introduction

Lignocellulosic feedstocks have attracted a lot of interest for the production of biofuels and other high added-value bio-based chemicals and materials. Production of biofuels from lignocellulosic biomass waste streams, such as agricultural or forestry residues, comprises the following steps: pretreatment, enzymatic saccharification, and microbial conversion of sugars to biofuels. Pretreatment is the first step towards overcoming the complexity and recalcitrance of lignocellulosic biomass, aiming to make cellulose susceptible to enzymatic hydrolysis [1]. The pretreatment process, aiming at removing lignin, is considered to be the costliest and most challenging part of the lignocellulose conversion scheme. Lignin, which is a polyphenolic polymer surrounds the cellulose and hemicellulose, and it is essentially responsible for making biomass highly recalcitrant to pathogens, microorganisms, and enzymes [2]. Hence, a pretreatment step is required in order to disrupt the carbohydrate–lignin complex and to allow for the hydrolytic enzymes to gain access to the carbohydrates [3,4,5]. Hydrothermal pretreatment, without the use of chemicals, efficiently degrades hemicelluloses and increases the biomass porosity, which, in turn, enhances enzymatic hydrolysis of the pretreated solids [6]. However, the lignin that cannot be removed via hydrothermal pretreatment is partly rearranged on the surface of the lignocellulosic biomass exhibiting an inhibitory effect on downstream enzymatic hydrolysis [7].

Organosolv pretreatment has attracted an increased research interest, as it offers an effective method to remove lignin with the use of organic solvents. Organosolv employs aqueous-organic solvent mixtures, with high solvent concentration (30–70%) at temperatures of 100–220 °C, with or without the addition of catalysts [8]. One of the main benefits of organosolv pretreatment is the isolation of high-quality lignin and high-purity lignin-free cellulose [9,10]. The lignin recovered is sulfur free, while the organic solvents used (ethanol, acetone, formic, and acetic acid, etc.) can easily be recovered which is a significant advantage for small scale biorefinery plants [11]. The addition of an organic solvent allows for better mass transfer and the dissolution of lignin [12], reducing its recondensation on the external surface area of the pulp [13]. In addition, organosolv pulps have bleachability and viscosity retention when compared to cellulose soda and kraft pulps [14].

For these reasons, there is significant research interest in investigating the best pretreatment method for lignocellulosic materials. Sequential hot water pretreatment for hemicelluloses depolymerization and organosolv delignification for the removal of lignin and the production of high purity pulps have been published [10,15,16]. These studies investigated the effect of the different pretreatment techniques on the physical and chemical properties of the pulps, together with the saccharification effect of the residual solid. The existence of a two-stage sequential pretreatment method has a negative impact in the economic feasibility of the process when compared with the one-stage pretreatment methods.

Typically, both hydrothermal and organosolv pretreatments are catalytically assisted with mineral acids and bases, such as NaOH, H_2_SO_4_, etc. Despite their wide use, there are some limitations; they are not environmentally friendly, they generate large quantities of acid wastes and require high energy inputs, thus increasing overall process cost [17,18,19]. For the above reasons, an effort is being made to replace or exclude highly corrosive mineral acids such as H_2_SO_4_. Use of milder acids, such as H_3_PO_4_ or even O_2_ combining organosolv and oxidation processes, are considered as interesting alternatives [20].

To make the production of ethanol economically viable and at the same time reduce the environmental impact of the process, the use of high solid concentration (high gravity—HG) during saccharification and fermentation can serve as a solution. The use of high solids concentration during saccharification can result in high glucose concentration in the broth and in turn in high ethanol production. It has been already argued that an ethanol content of at least 4% *w*/*w* is required for an economically feasible ethanol distillation [21]. Moreover, HG processes are advantageous from a water economy point of view [22]. Despite the obvious advantages of HG processes, they also present several challenges during their implementation. The high solids content create a very viscous material, practically without any free water, which is hard to mix and pump, leading to insufficient mass and heat transfer [23]. Various alternatives have been proposed to overcome these issues and achieve efficient saccharification of lignocellulosic biomass under HG conditions, such as fed-batch hydrolysis [24]. Towards this direction, Luleå University of Technology (LTU) group has previously developed and implemented a free-fall mixing reactor that was successfully used for the saccharification of various lignocellulosic materials, such as sweet sorghum bagasse [25], food waste [26], corn stover [27], wheat straw [28], and beech wood [20] at high solids content prior to ethanol fermentation. Other groups have also developed high gravity processes, successfully fermenting steam pretreated spruce to ethanol [29] or beechwood to biobutanol and dicarboxylic acids in a Terrafors reactor [30].

In this work, different organic solvents were tested for the pretreatment of beechwood sawdust in an effort to efficiently delignify the biomass. The pretreatment conditions were optimized by studying the effect of the organic solvent, concentration, and type of acidic catalyst. The aim was to maximize lignin removal, while achieving high cellulose purity and recovery in the resulting pulps. The pulps were tested for their potential in enzymatic release of glucose. The materials demonstrating the highest saccharification yields were used in HG saccharification and fermentation at a solid content of 20 wt.%. Saccharification was done in a HG custom made reactor and it resulted in the production of an aqueous solution containing up to 8.0 wt.% ethanol in the subsequent fermentation. In addition, the removed lignin was easily recovered via solvent distillation and precipitation, and found to be potentially of high quality, being suitable for further conversion towards added value products.

## 2. Results and Discussion

### 2.1. Effect of the Type of Organic Solvent

Table 1 presents the experimental conditions of all runs conducted, while Table 2 presents the lignin, cellulose, and hemicellulose content of the pretreated pulp, together with the recoveries of each individual component into the pretreated pulps. It should be noted that, in some cases, the recoveries of the constituents are calculated at above 100%, due to the experimental errors of the analytical methods.

**Figure 1:**
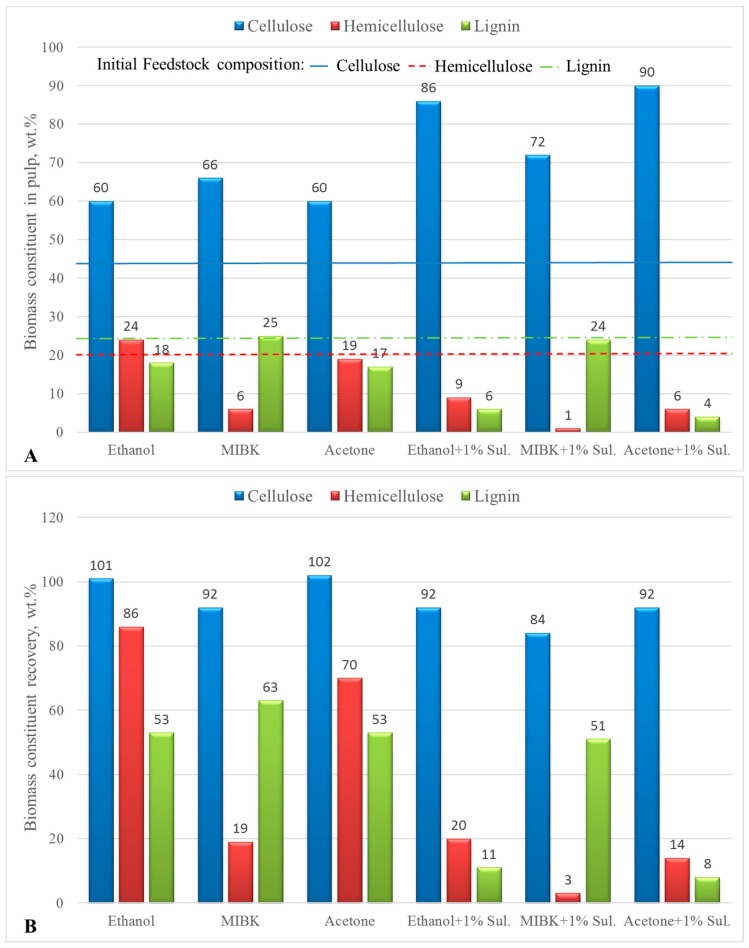
Pulp compositions (**A**) and biomass constituents’ recoveries (**B**) in solid pulps in batch autoclave runs at 175 °C, 1 h reaction time, LSR = 10, effect of organic solvents without and with use of 1 wt.% H_2_SO_4_, data labels have been rounded for clarity of presentation.

Varying degrees of delignification were achieved depending on the solvent used and the presence or absence of acids that act as catalysts. Figure 1 presents graphically the pulp compositions and biomass constituents’ recoveries when employing different organic solvents with and without homogeneous acidic catalysis.

Organosolv pretreatment was found to be very efficient in the pretreatment of beech wood biomass, as high cellulose low lignin content was achieved in all the treatment conditions. In some cases, the cellulose content exceeded 80 wt.% (runs 2, 7, 8, 9, and 11). Accordingly, the lignin content was very low, ranging from 4.2 to 10.7 wt.%. The pairs of runs 1–2, 3–4, and 6–7 employed different organic solvents, specifically, ethanol (EtOH), methyl- isobutyl- ketone (MIBK), and acetone (AC), without and with the use of 1 wt.% H_2_SO_4_ as catalyst. EtOH and AC are water miscible solvents, typically used in organosolv processes. MIBK is water immiscible, forming a biphasic system with water, which on one hand, can impact the fractionation efficiency of the system, but on the other hand, can significantly simplify the separation process of the organic lignin rich fraction from the aqueous carbohydrates rich fraction. MIBK has been used in biphasic systems for production of chemicals from biomass [31], as a co-solvent during fractionation of organosolv lignin in single phase systems [32,33] and as an extracting agent for the isolation of lignin from liquors rich in lignin and hemicellulose [34].

Regardless of the solvent used, the use of H_2_SO_4_ resulted in lower hemicellulose and lignin content and higher cellulose content in all of the pulps. Clearly, the hydrolyzing effect of the catalyst allowed for easier and more effective hemicellulose hydrolysis and removal. This, in turn, made the removal of lignin easier since it is closely connected to hemicellulose through a variety of bonds, such as ether and hydrogen bonds [35,36]. Among the three solvents used, both AC and EtOH proved to be effective in delignifying the biomass. AC was slightly more effective probably due to its higher solvent strength. On the other hand, MIBK was not as effective in delignifying the biomass. Compared to AC and EtOH, MIBK has a Hildebrand solubility parameter of 8.4, which is lower than typical lignin solvents, which is in the 10.5–12.5 range [32]. In addition, MIBK is not soluble in water, making this a two liquid phase reaction system. MIBK’s insolubility in water is responsible for its poor performance, however it is this property that makes it very interesting for use as delignifying agent. Since the organic phase, which contains the dissolved lignin, and the aqueous phase, which contains the hemicellulose hydrolysate, can be very easily separated by spontaneous phase separation, this simplifies the separation process, and in turn, reduces the energy demands for lignin recovery. Hence, the 50% delignification degree (DD) achieved, although low, is satisfactory enough to justify further investigation in future work. Teng et al. [37] used the H_2_O/MIBK biphasic system successfully to delignify different biomasses such as corn cob and rice straw. They found that the use of acidic ionic liquids (IL) was significantly more efficient when compared to the use of mineral acids. Use of H_2_SO_4_ achieved a DD of 61.5%, while the use of the IL [C_4_H_8_SO_3_Hmim]HSO_4_ resulted in a DD of 76.3%. Pretreatment without the use of any catalyst resulted in poor delignification with a DD of 24% for corncob. They attributed the lower efficiency of the mineral acids to their miscibility in MIBK, which resulted in a reduction of their actual concentration in the aqueous solution, lowering their catalytic efficiency. In our work, use of mineral acids in the case of MIBK increased the DD from 37.2% to 49.5%, which is in accordance to the findings by Teng et al. [37]. Another interesting note is that in runs 3 and 4 where MIBK is used, there is a significant reduction of the hemicellulose that is retrieved in the solid pulp without H_2_SO_4_ (19 wt.%) and with H_2_SO_4_ (3.4 wt.%) when compared to EtOH (85.8 and 20.5 wt.%, respectively) and AC (70 and 13.6 wt.%, respectively). The immiscibility of the MIBK with water resulted in the stronger solvent power and hydrolysis effect of the water towards the biomass hemicellulose. In contrast, EtOH and AC that are water miscible act as antisolvents, in part reducing the hydrolysis achieved by H_2_O. This is also validated by the cellulose recovery in the pulps, which in the case of runs 3 and 4 with MIBK drops to 92.1 and 83.8 wt.% without and with H_2_SO_4_ respectively. Cellulose, which is much more recalcitrant compared to hemicellulose [31], is not that affected, but part of it is solubilised in the aqueous fraction, especially when H_2_SO_4_ is employed. Apparently, the use of one phase systems with EtOH and AC results in even lower solubilisation of cellulose, hence most of it is recovered in the solid pulp.

### 2.2. Effect of Catalyst Type and Concentration

**Figure 2:**
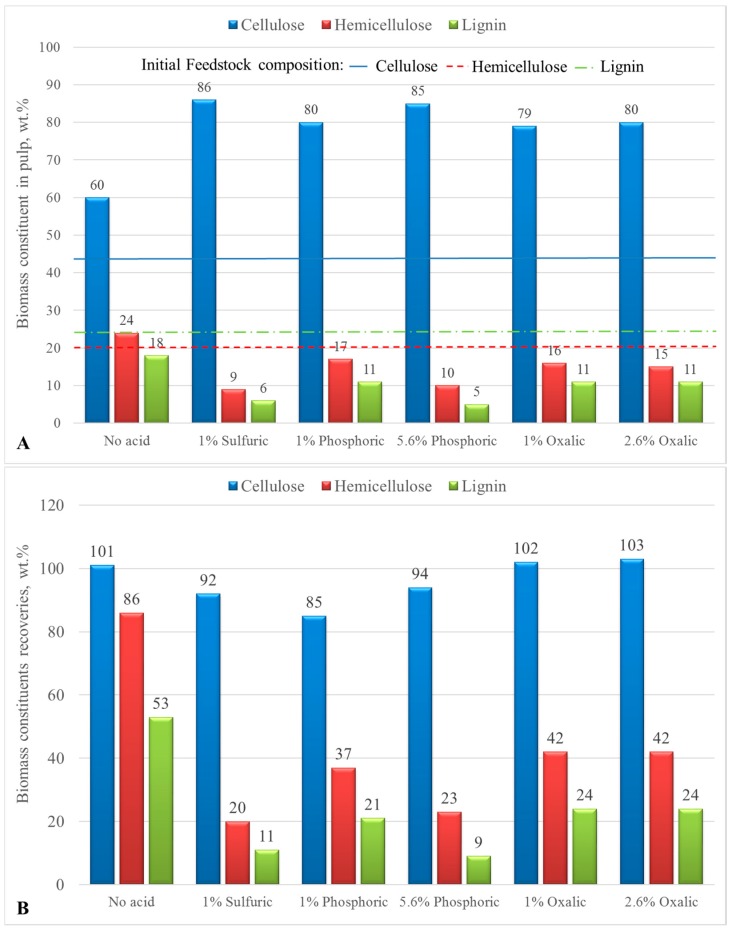
Pulp compositions (**A**) and biomass constituents recoveries (**B**) in solid pulps in batch autoclave runs at 175 °C, 1 h reaction time, LSR = 10, effect of homogeneous catalysis, data labels have been rounded for clarity of presentation.

Runs 8–11 along with runs 1 and 2 aimed at understanding the effect that homogeneous catalysis can have on the removal of lignin and the depolymerization and hydrolysis of hemicellulose in the bid to produce a high cellulose pulp. For this purpose, three different types of acids were investigated and their effect on the composition of the pulps is graphically presented in Figure 2. H_2_SO_4_ was tested as a base case scenario, since it is the most used acid for biomass pretreatment [38]. H_3_PO_4_ was tested as an inorganic acid alternative. Its main advantages are the fact that it is much less corrosive, easier to recycle, and can yield more amorphous cellulose pulp [39]. Oxalic acid was tested as an organic acid alternative. Dicarboxylic acids exhibit some advantageous characteristics, such as controlled stepwise acidity, biodegradability, and diminished corrosivity. In addition, they can be produced from bio-based and renewable resources, making them particularly attractive catalysts for biomass conversion [40].

Comparing run 1 and 2, the use of H_2_SO_4_ at 1 wt.% on dry biomass basis as catalyst has a pronounced effect, increasing the removal of hemicellulose and lignin from 14 and 46 to 80 and 89%, respectively. As expected, it enhanced hemicellulose hydrolysis, which also facilitated the removal of lignin, since these two components are connected via ether bonds, removing one can significantly boost the removal efficiency of the other. Both phosphoric and oxalic acids were also tested as catalysts. Run 8 and 10 employed 1 wt.% of each acid on a biomass basis, while run 9 and 11 used 5.6 and 2.6 wt.% of phosphoric and oxalic, respectively. This was done in order to reach the same pH as in the case of 1 wt.% H_2_SO_4_, so as to test the three different catalysts at the same severity. Phosphoric acid proved to be quite efficient in enhancing hemicellulose hydrolysis and lignin removal, at the 5.6 wt.% addition it was marginally better when compared to H_2_SO_4_ for delignification. The addition of oxalic acid also increased the efficiency of delignification when compared to the treatment without acid catalysis. However increasing its concentration had no further effect. Stein et al. [41] achieved delignification using oxalic acid as catalyst in a water/2-methyltetrahydrofuran (2-MTHF) biphasic system. Oxalic acid has been previously used to depolymerize the hemicellulosic part of biomass [41], leaving the cellulosic crystalline part intact even at temperatures as high as 180 °C [42]. The above is in accordance with our work. Cellulose recovery in the solid pulp was 100% when oxalic acid was added, however hemicellulose recovery in solid form dropped from ~86% of initial hemicellulose when no oxalic acid was used to ~42% with oxalic acid catalysis. Lignin was also successfully removed, its recovery in the solid pulp dropped from 53.5% to ~24% (run 1, 10, 11, in Table 1).

### 2.3. Pulp and Lignin Quality

Apart from the composition of the resulting pulps, their crystallinity index (CrI) was determined as an attempt to evaluate the effect of the pretreatment on the pretreated solids and their potentials for enzymatic saccharification. Table 3 presents the CrI of all the produced pulps.

**Figure 3:**
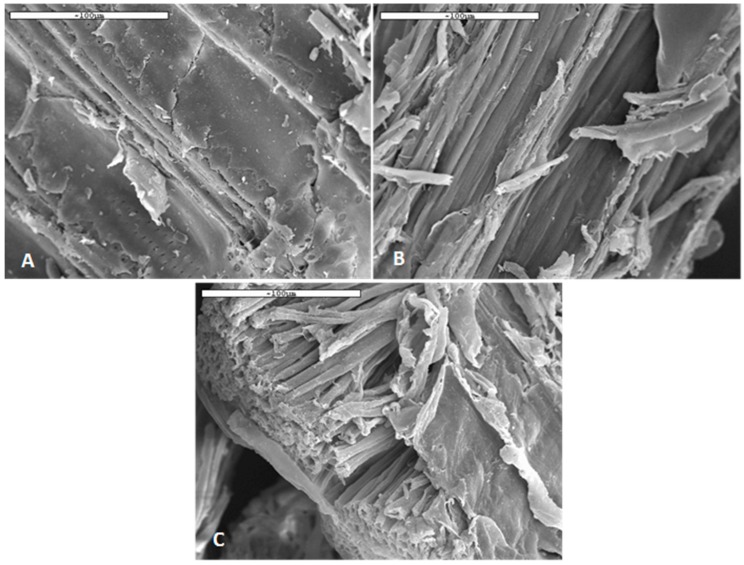
Scanning electron microscopy (SEM) images, bar scale of 100 μm (**A**) untreated beechwood, (**B**) AC-1%H_2_SO_4_, and (**C**) EtOH-5.6%H_3_PO_4_.

As expected, there is an overall trend that resulted in the increase of the CrI as the cellulose content in the pulp increased due to the inherent crystallinity of the cellulosic part of the biomass. Run No. 1, for example, had cellulose content of 60% corresponding to a CrI of 68.8%, while runs 7 and 9 with increased cellulose contents of 89 and 85% had CrI at around 78%. In addition, it is noted that it is the presence of hemicellulose rather than lignin in the pulp that lowers the CrI. Pulps with high hemicellulose content had lower CrI due to the hemicellulose amorphous regions. Figure 3 presents SEM images of the initial biomass and pulps retrieved from run 7 and 9, which employed H_2_SO_4_ and H_3_PO_4_, respectively.

It appears that the removal of lignin and hemicellulose results in the partial change in the fiber morphology. Untreated beechwood (Figure 3A) has a relatively smooth surface, while AC-1%H_2_SO_4_ and EtOH-5.6%H_3_PO_4_ pulps have rougher surface. Especially in the case of EtOH-5.6%H_3_PO_4_, the pulp appeared to be partially defibrilated and individual cellulose fibers were exposed (Figure 3C). The surface area of the pulps was slightly increased when compared to the untreated beechwood. More specifically, untreated beechwood had surface area of 0.27 m^2^/g, while for pulps that are produced from run 7 and 9, this increased to 1.18 m^2^/g and 1.08 m^2^/g, respectively. This is a small increase in surface area but has been found to positively affect the enzymes’ efficiency. Arantes et al. concluded that the topology/porosity of the pulp can limit protein penetration into the microfibril pores of the pulp, and hence affect the enzyme efficiency [43]. This is in agreement with the findings of Thygesen et al. who showed that the enzymes first penetrated into the porous regions of the pulp, and subsequently hydrolysed the cellulosic parts towards mono and oligomeric sugars [44].

**Figure 4:**
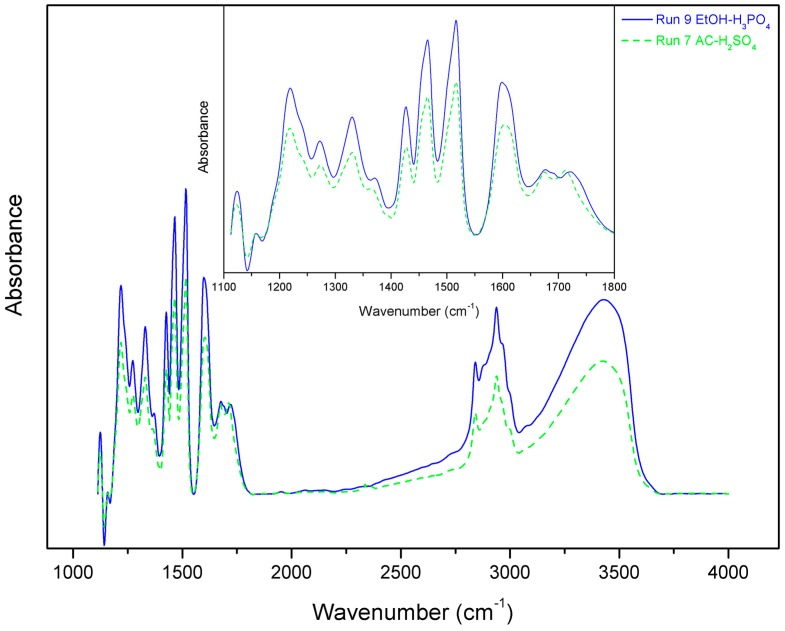
Fourier Transform Infrared Spectroscopy (FTIR) spectra of lignins retrieved from beechwood delignification from run 7 (AC-1%H_2_SO_4_) and 9 (EtOH-1%H_3_PO_4_).

Lignins were retrieved from all runs and some selected samples were analysed via NREL to evaluate their purity. The lignins from run 7 and 9, which were found to be the most suitable for biomass delignification, were found to have very high lignin content at >94.5 wt.% and 92 wt.% purity, respectively. Lignin from run 7 had 0 wt.% cellulose content and only 0.8 wt.% hemicellulose content. For comparison, lignin from run 6 had lignin content of around 89 wt.% and hemicellulose content around 4.2 wt.%. The lack of an acid catalyst in the case of run 6 led to the sedimentation of some hemicellulose oligo- and poly- saccharides. The use of the severe H_2_SO_4_ in the case of run 7 hydrolyzed hemicellulose to such an extent that none was retrieved in the solid fraction of lignin. Run 9, on the other hand, had 2 wt.% and 1.8 wt.% cellulose and hemicellulose content, respectively. The milder acidity of H_3_PO_4_ was enough to solubilize a small part of cellulose and leave some hemicellulose intact, so as to receive it in the solid lignin. Overall, all of the lignins retrieved were very pure and well fractionated. Finally, the lignins from run 7 and 9 were also analysed via FTIR (Figure 4).

From the spectra, it appears that the delignification treatment did not degrade the recovered lignin. The FTIR graphs have peaks at characteristic wavelengths below 1500 cm^−1^, corresponding to guaiacyl, syringyl, and some methyl- and methylene- side chains that are typically found at 1385, 1420, and 1463 cm^−1^ [45]. Wavelengths at 1216, 1271, and 1328 cm^−1^, corresponding to stretching of C–C and C–O bonds in guaiacyl oligomers and condensed syringyl and guaiacyl rings typical of hardwood lignin are also detected [46], suggesting that the structures of the lignins remain intact. This is a very important finding, since this pure lignin product, which is easily recovered from the solvent mixture, could be upgraded to high value chemicals towards the establishment of a holistic biorefinery approach.

### 2.4. Enzymatic Saccharification of Pretreated Pulps

To evaluate the potential of the pretreated pulps as raw materials for ethanol production, their susceptibility to enzymatic saccharification was assessed under low solids content. Table 4 presents the cellulose conversion after 24 and 48 h of enzymatic saccharification. The numbers in parentheses in the 24 h column indicate how much of the total glucose production occurred in the first 24 h, which is an important parameter and is indicative of the conversion speed.

**Figure 5:**
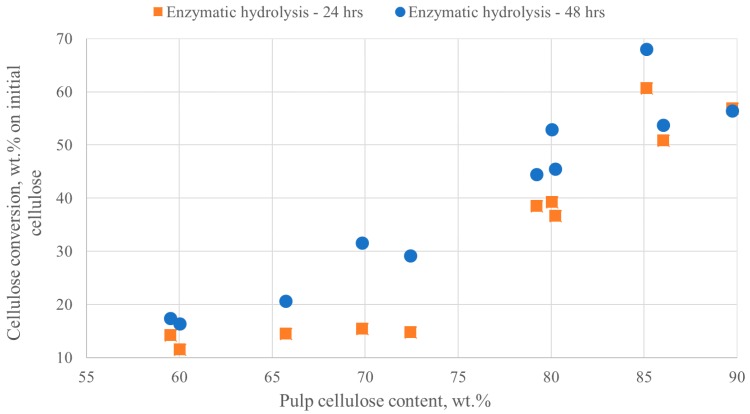
Cellulose conversion to glucose via enzymatic hydrolysis at 24 and 48 h vs pulp cellulose content.

An overall trend is noted where the higher the cellulose content of the pulp, the higher the cellulose conversion percentage was achieved (Figure 5). This is attributed to the lower lignin content of the high cellulose content pulps. Lignin has been known to have significant impact on the enzymes used for cellulose hydrolysis, inhibiting the depolymerisation of cellulose and the production of monomeric sugars [2]. In addition, some interesting observations can be deduced from the combination of Table 4 and Figure 5. More specifically, run 2 and 7–11, where homogeneous acidic catalysis was employed, produced pulps that were enzymatically hydrolysed to glucose easier (higher conversion after 48 h), but also more rapidly (higher % of conversion in first 24 h). Run 2 and 7 have the highest conversion rates; ~95 and 100% of overall cellulose to glucose conversion occurs in the first 24 h, respectively. This can be attributed not only to the high DD achieved, but also to a partial depolymerization of the cellulose to lower molar mass cellulose that can be enzymatically hydrolysed more rapidly. Run 9, where 5.6 wt.% H_3_PO_4_ was used, had the highest conversion of cellulose at 24 and 48 h, higher than that of run 7 at roughly the same lignin content. Work in the literature suggests that treating biomass with concentrated H_3_PO_4_ results in the swelling of the fibres and the reduction of the cellulose crystallinity [47,48]. In our work, the CrI increased as a consequence of the increased cellulose content of highly delignified pulp. Pulps produced with the aid of H_2_SO_4_ or H_3_PO_4_ catalysis had no significant differences in the CrI at similar cellulose and lignin contents (runs 2, 7, 9). Sathitsuksanoh et al. treated biomasses with concentrated H_3_PO_4_ and found that the CrI values varied greatly, depending on several parameters, such as measurement techniques, calculation approaches, and sample drying conditions. They concluded that the effects of CrI data obtained from dried samples on enzymatic hydrolysis should be interpreted with caution. On the other hand, they suggested that increase of the fibres surface area through lignin and hemicellulose removal and disruption of the hydrogen bonds found in crystalline cellulose could significantly increase the hydrolysis rates and efficiencies [49]. Hence, a possible explanation for the hydrolysability of pulps produced with H_3_PO_4_ assisted catalysis is the disruption in part of hydrogen bonding, which is not necessarily depicted as a reduction in the CrI. Enzymatic hydrolysis proved to be dependent mostly on cellulose and lignin content and was irrelevant of the CrI. Lignin, which has been found to be a major inhibitor in cellulose saccharification should therefore be removed to achieve high glucose production [50,51].

### 2.5. High Solids Hydrolysis and Fermentation

**Figure 6:**
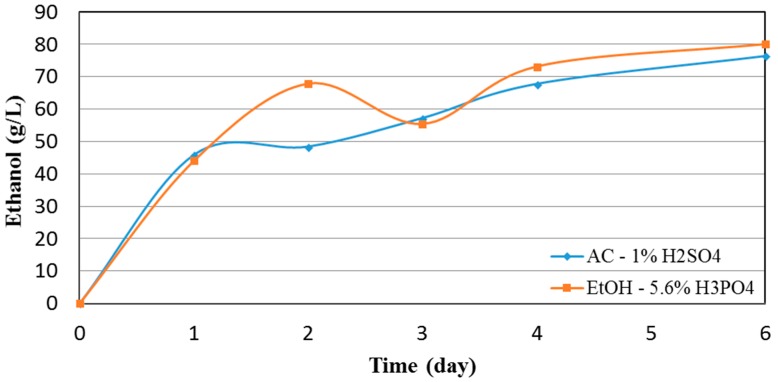
Ethanol concentration in high solids simultaneous saccharification and fermentation (SSF), an 8 h hydrolysis step preceded the SSF.

Based on the results from the saccharification at low solid content, two different delignified pulps, specifically from run 7 and 9, which employed AC with 1 wt.% H_2_SO_4_ and EtOH with 5.6 wt.% H_3_PO_4_, respectively, were selected for evaluation under high solids hydrolysis and fermentation towards ethanol. The pulps from run 7 and 9 were found to have the highest DD, lowest lignin content, and over 90 wt.% cellulose recovery in the solid pulp. They were thus deemed suitable for high solids simultaneous saccharification and fermentation (SSF). As noted in the Methods section, the liquefaction/saccharification duration was 8 h at an enzyme loading of 8.4 mg/g_solids_. After 8 h of pre-liquefaction/saccharification, the concentration of glucose was 63.8 g/L and 74.7 g/L, corresponding to 32.1% and 39.5% cellulose saccharification for the H_2_SO_4_ and H_3_PO_4_ assisted runs, respectively. Efficient glucose production in the first 8 h meant that ethanol concentrations higher than 40 g/L could be reached; a required minimum for downstream low-cost distillation [17]. Figure 6 presents the evolution of ethanol concentration for a six-day period for both delignified pulps.

Both pulps reached the 40 g/L ethanol concentration threshold in less than 24 h of SSF. The AC–H_2_SO_4_ delignified pulp produced slightly more EtOH the first 24 h reaching a concentration of ~46 g/L. Afterwards, the ethanol production gradually leveled off to a final concentration of 76.3 g/L after six days of SSF, which is equal to approximately 75% of the maximum theoretical ethanol yield that could be attained for the cellulose content of the pulp. The EtOH-H_3_PO_4_ (pulp No. 9), on the other hand, had a slightly lower production rate in the first 24 h, however it retained its high production rate for up to 48 h, reaching an ethanol concentration of 68.7 g/L after the first 48 h of SSF. After six days of SSF, the ethanol concentration reached a maximum of 80 g/L, which is equal to approximately 83% of the maximum theoretical ethanol yield. Pulp No. 7 demonstrated a slightly higher productivity during the first 24 h of fermentation. This difference in the initial ethanol productivity, can be attributed to a minor inhibition of the fermentation process by the higher initial glucose concentration; behavior that has also been observed elsewhere [27]. Pulp No. 9, which used H_3_PO_4_, had a steadier fermentation rate for up to 48 h. Even though its cellulose content was slightly lower when compared to pulp No. 7, it achieved higher final ethanol concentration. As explained above, the H_3_PO_4_ may have disrupted in part the hydrogen bonds allowing for more efficient cellulose hydrolysis and consequently fermentation towards ethanol. The HG results are in good agreement with the initial enzymatic hydrolysis evaluation runs, where pulp No. 7 was found to quickly reach its maximum conversion in the first 24 h, while pulp No. 9 gave higher overall conversion in the 48 h period. Table 5 summarizes some of the work that has been done in HG SSF of different types of lignocellulosic feeds for the production of ethanol. The ethanol concentration of 80 g/L, as reported in our work, is one of the highest achieved in the literature.

## 3. Materials and Methods

### 3.1. Raw Materials

Commercially available beechwood sawdust with particle size 150–500 μm (Lignocel^®^ HBS 150–500) and moisture content 8 wt.% was used as biomass feedstock. It was handled, as described by Kalogiannis et al. [45].

### 3.2. Strains and Enzymes

The *Saccharomyces cerevisiae* strain Ethanol Red^®^ was used as fermenting microorganism during the current work. This specific strain was developed by Fermentis (Marcq-en-Barœl, France) for industrial fuel ethanol production, and therefore it exhibits high ethanol tolerance, making it suitable for use in HG fermentation processes. The commercial enzyme solution Cellic^®^ CTec2 from Novozymes A/S (Bagsværd, Denmark) was used for the saccharification trials under low solids content and at HG conditions. The protein content of the enzyme solution was 100 mg/mL, as determined by using the Bradford assay [64]. All the other chemicals and reagents were of analytical grade.

### 3.3. Organosolv Pretreatment

Organosolv pretreatment of Lignocel was performed in metallic cylinders of 2.5 L size, which were placed in an air-heated multidigester apparatus [27] at 175 °C for 60 min. During the pretreatment, 110 g of biomass were mixed with 1.1 L of solvent-aqueous mixture. The following solvents were tested: ethanol, acetone, and methyl-isobutyl-ketone at a content of 60% *v*/*v* (with the acetone to be also tested to an acetone content of 25% *v*/*v*) with or without the addition of sulfuric acid (1 wt.% on dry biomass) as acidic catalyst. Replacement of sulfuric acid with phosphoric acid and oxalic acid was also tested with ethanol as the solvent. In that case, the concentration of the acid catalysts was either similar to sulfuric acid (1 wt.% on dry biomass) or was fixed to achieve the same pH as the sulfuric acid during the pretreatment (phosphoric acid, 5.6 wt.% on dry biomass; oxalic acid, 2.6 wt.% on dry biomass). At the end of the pretreatment, the pretreated solids were separated from the pretreatment liquor by vacuum filtration, washed with the same solvent used during the pretreatment, air dried, and stored until further use. The weight of the pretreated solids was measured to determine biomass solubilization and the composition of the solids was determined, as described in the *Analytical Methods* section.

The pretreatment liquor was collected and the solvent was evaporated (when ethanol and acetone were used) under vacuum in order to reduce lignin solubility. Lignin was then separated from the liquid by centrifugation (14,000 rpm, 29,416× g, at 4 °C for 15 min), and finally air-dried [53]. When MIBK was used as solvent, a different lignin isolation process was followed. MIBK is water immiscible at room temperature, resulting in phase separation with the lignin being recovered in the solvent phase. The solvent was then evaporated under vacuum, leading to the recovery of the solid lignin.

All of the experimental conditions are presented in Table 1. The resulting pulps were dried and weighed, while the original biomass and the resulting pulps were analysed by the NREL method to determine (see analytical methods paragraph) cellulose, hemicellulose, and lignin content. The delignification degree (DD) can be calculated as 100%-lignin recovery (%).

### 3.4. Enzymatic Saccharification Trials

The pretreated solids were assessed for their enzymatic saccharification yields under a solid content of 2 wt.% in 50 mM citrate buffer (pH 5). The enzyme load was 8.4 mg/g_solids_ of the commercial enzyme solution Cellic^®^ CTec 2. Sodium azide at a concentration of 0.02 wt.% was added in the solution to prevent microbial contaminations. Incubation took place in 2 mL Epperdorf tubes containing 1.5 mL of the solution in ThermoMixer C (Eppendorf, Hamburg, Germany) at 50 °C and 1200 rpm for 48 h. Samples were withdrawn at 0 h, 24 h, and 48 h, and analyzed for glucose concentration. All of the enzymatic hydrolysis trials were performed in duplicates. The enzymatic saccharification yield was expressed as the percentage of cellulose converted to glucose and was calculated according to the following equation:

$$Saccharification\;yield=\left(\frac{C_{glucose}\times V_{liquid}\times 0.90}{m_{solids}\times x_{cellulose}}\right)\times 100$$

where *C_glucose_* is the concentration of glucose, *V_liquid_* is the volume of the liquid during the trials, 0.90 is the correction factor for the conversion of glucose to cellulose, *m_solids_* is the mass of the dry solids, and *x_cellulose_* is the cellulose content of solids.

### 3.5. High Gravity Saccharification and Fermentation

The two most promising materials were further subjected to high gravity saccharification and fermentation trials. Saccharification took place at a freefall mixing saccharification reactor, as previously described [25]. More specifically, the dry material content used was 20 wt.% in 50 mM citrate buffer with an enzyme load of 8.4 mg/g_solids_. Saccharification took place at 50 °C for 8 h. At the end of the saccharification the slurry was collected and supplemented with nutrients for the yeast growth at a final concentration of 1 g/L yeast extract, 0.5 g/L (NH_4_)_2_HPO_4_, and 0.025 g/L MgSO_4_·7H_2_O. The fermentation was initiated by inoculation with *S. cerevisiae* suspension (that was grown overnight at YPD media at 35 °C and 180 rpm) to achieve an initial dry cell weight concentration of 1 g/L. Incubation was carried out at 35 °C and 120 rpm, and the samples were withdrawn daily, diluted, filtered through a 0.2 μm syringe filter, and analyzed for ethanol. The fermentations were performed in duplicates.

### 3.6. Analytical Methods

The cellulose, hemicellulose, lignin and ash contents of lignocellulosic biomass were determined, according to the procedures provided by National Renewable Energy Laboratory (NREL; Golden, CO, USA) [65]. The sugars were analysed at a high pressure liquid chromatography (HPLC) apparatus, coupled with a refractive index detector equipped an Aminex HPX-87P column (Bio-Rad, Hercules, CA, USA). Analysis performed at 85 °C, with ultrapure water as mobile phase at a flow rate of 0.6 mL/min. Ethanol produced during the SSF was analysed by the same HPLC apparatus equipped with an Aminex HPX-87H (Bio-Rad, Hercules, CA, USA) chromatography column. The column was kept at 40 °C and the mobile phase was 5 mM sulphuric acid in degassed HPLC grade water at a flow rate of 0.6 mL/min.

Fourier Transform Infrared Spectroscopy (FTIR) (Nicolet 5700, Thermo Electron Corporation, Waltham, MA, USA) analysis was employed for further characterization of the lignin samples’ structure. Details may be found elsewhere [46]. X-ray Diffraction analysis was done on a Siemens D500, copper ray with a Nickel filter (λ = 15,406 Å, voltage 40 KV, intensity 30 mA) (Bruker, Wien, Austria). The angle 2*θ* was between 5° and 50° with a step 0.04 and step time 2 s. Surface area of the pulps was measured on a Micromeritics Tristar 3000 (Micromeritics, Norcross, GA, USA) via the BET method after outgassing the biomass samples at 25 °C for 72 h. Scanning electron microscopy (SEM) images were obtained on a Jeol JSM-6300 microscope (Jeol, Peabody, MA, USA).

For the determination of the surface area (BET method), pore volume, and pore size distribution (BJH method) of the catalyst samples, N_2_ adsorption/desorption measurements were carried out at −196 °C, using an Autosorb-1MP Automatic Volumetric Sorption Analyzer (Quantachrome, Boynton Beach, FL, USA).

## 4. Conclusions

In the present work, the efficiency of organosolv pretreatment on lignin and hemicellulose removal and its effect on the downstream biochemical conversion of the solid pulp to ethanol were evaluated. A hardwood feedstock, more specifically beechwood, was treated with mixtures of water and different organic solvents, namely AC, EtOH, and MIBK. In addition, the effect of homogeneous catalysis was investigated. Sulfuric, phosphoric, and oxalic acids were tested at different concentrations and their effect on the DD and the hydrolysability and fermentability of the resulting pulps was evaluated. Both AC and EtOH, which are water miscible, were found to be very efficient in removing lignin and hemicellulose from the initial feedstock. Both were able to remove almost 50% of the lignin found in the feedstock. MIBK, on the other hand, behaved poorly due to its non-miscibility in water. Use of sulfuric acid as catalyst significantly improved the DD; more than 90% of initial lignin was removed and pulps with high cellulose content (>85%) were produced. Phosphoric and oxalic acid were used as alternative catalysts and were both found to enhance lignin removal. In the case of phosphoric acid, partial defibrillation and exposure of the cellulose fibrils was also noted. Moreover, the lignin retrieved from the solvent system was found to be intact and of high purity and quality making it a valuable potential feedstock for production of bio-based chemicals and materials. High gravity SSF at 20 wt.% solids yielded highly concentrated ethanol solutions (8 wt.%), which is one of the highest reported in the literature for beechwood feedstock and stresses the potential of combining organosolv pretreatment with high solids fermentation on the basis of a biorefinery approach.

## Figures and Tables

**Table 1:** Experimental conditions for the organosolv pretreatment.

Run No. *	Solvent	Solvent, vol.%	Catalyst, wt.% on Dry Basis
**1**	Ethanol	60	-
**2**	Ethanol	60	H_2_SO_4_, 1.0%
**3**	MIBK	60	-
**4**	MIBK	60	H_2_SO_4_, 1.0%
**5**	Acetone	25	-
**6**	Acetone	60	-
**7**	Acetone	60	H_2_SO_4_, 1.0%
**8**	Ethanol	60	H_3_PO_4_, 1.0%
**9**	Ethanol	60	H_3_PO_4_, 5.6%
**10**	Ethanol	60	C_2_H_2_O_4_, 1.0%
**11**	Ethanol	60	C_2_H_2_O_4_, 2.6%

* Reaction temperature: 175 °C, reaction time: 60 min, liquid to solid (LSR) ratio: 10.

**Table 2:** Biomass constituents pulp content and % retrieved in the solid pulp.

Run No.	Cellulose (%)	Hemicellulose (%)	Lignin (%)	Cellulose Retrieved (%)	Hemicellulose Retrieved (%)	Lignin Retrieved (%)
Initial Biomass *	43.1	20.2	24.2	-	-	-
**1**	60.0	23.8	17.8	101.5	85.8	53.5
**2**	86.0	9.0	5.7	92.5	20.5	11.0
**3**	65.7	6.4	25.2	92.1	19.0	62.8
**4**	72.4	1.4	24.5	83.8	3.4	50.6
**5**	69.8	9.1	19.9	94.4	26.2	47.9
**6**	59.5	19.1	17.2	102.3	70.0	52.7
**7**	89.7	6.3	4.2	91.7	13.6	7.6
**8**	80.2	16.6	10.9	85.0	37.5	20.6
**9**	85.1	10.0	4.6	93.6	23.3	9.1
**10**	79.2	15.5	10.6	102.0	42.4	24.2
**11**	80.0	15.2	10.7	103.2	41.7	24.5

* Untreated Lignocel extractives are 9.1%.

**Table 3:** Crystallinity index (CrI) of pretreated pulps.

Run No.	Crystallinity Index CrI (%) *
**1**	68.8
**2**	77.5
**3**	74.1
**4**	78.2
**5**	74.4
**6**	69.0
**7**	78.1
**8**	75.1
**9**	77.3
**10**	73.2
**11**	72.9

* Standard deviation for CrI was ± 1.3%.

**Table 4:** Enzymatic hydrolysis to glucose at 24 and 48 h.

Run No.	24 h * (wt.% on Feed Cellulose)	48 h (wt.% on Feed Cellulose)
**1**	11.6 (70.0)	16.5
**2**	50.9 (94.6)	53.8
**3**	14.6 (70.5)	20.7
**4**	14.9 (50.8)	29.3
**5**	15.5 (48.9)	31.7
**6**	14.4 (82.3)	17.5
**7**	57.0 (100.9)	56.5
**8**	36.8 (80.6)	45.6
**9**	60.8 (89.3)	68.1
**10**	38.7 (86.8)	44.6
**11**	39.3 (74.2)	53.0

* numbers in parentheses depict the percentage of the amount of cellulose hydrolyzed to glucose in 24 h to the amount hydrolyzed in 48 h.

**Table 5:** Work found in the literature on high gravity (HG) SSF for ethanol production.

WIS (%)	Material	Pre-Treatment	Enzyme Loading	Ethanol (g/L)	Time (h)	Reference
**20**	Beechwood	Organosolv with acetone and sulphuric acid	8.4 mg/g	76.3	144	Current work
**20**	Beechwood	Organosolv with ethanol and phosphoric acid	8.4 mg/g	80	144	Current work
**20**	Beechwood	Acetone/water oxidation	8.4 FPU/g	75.9	120	[20]
**36**	Bermudagrass	Phosphoric acid-acetone	25 FPU/g cellulose	56.1	96	[52]
**20**	Birch	Hybrid organosolv–steam explosion	18.5 FPU/g	80	192	[53]
**20**	Birch	Steam pre-treated	20 FPU/g	14.4	144	[54]
**20**	Corn stover	Steam explosion	17.7 FPU/g	59.8	192	[55]
**20**	Eastern redcedar	Acid bisulfite	46 FPU/g glucan	52	42	[56]
**15**	Eucalyptus	Organosolv	20 FPU/g	42	72	[57]
**20**	Rapeseed straw	Dilute acid	15 FPU/g	39.9	24	[58]
**25**	Pine	Sulfite	15 FPU/g	82	24	[59]
**20**	Spruce	Steam pre-treated	22.5 FPU/g	40	96	[60]
**10**	Spruce	Steam pre-treated	30 FPU/g glucan	45	100	[61]
**10**	Spruce	Steam pre-treated	20 FPU/g	45.8	96	[62]
**25**	Wheat straw	Steam explosion	15 FPU/g	58.6	80	[63]
